# Prior National Drug Abuse Treatment Clinical Trials Network (CTN) opioid use disorder trials as background and rationale for NIDA CTN-0100 “optimizing retention, duration and discontinuation strategies for opioid use disorder pharmacotherapy (RDD)”

**DOI:** 10.1186/s13722-021-00223-z

**Published:** 2021-03-06

**Authors:** Matisyahu Shulman, Roger Weiss, John Rotrosen, Patricia Novo, Elizabeth Costello, Edward V. Nunes

**Affiliations:** 1 New York State Psychiatric Institute and Columbia University Irving Medical Center, 1051 Riverside Drive, New York, NY 10032 USA; 2 McLean Hospital and Harvard Medical School, Boston, USA; 3 New York University Grossman School of Medicine, New York, USA

**Keywords:** Opioid use disorder, Buprenorphine, Naltrexone, Clinical trials

## Abstract

Opioid use disorder continues to be a significant problem in the United States and worldwide. Three medications—methadone, buprenorphine, and extended-release injectable naltrexone,— are efficacious for treating opioid use disorder (OUD). However, the utility of these medications is limited, in part due to poor rates of retention in treatment. In addition, minimum recovery milestones and other factors that influence when and whether individuals can safely discontinue medications are unknown. The National Drug Abuse Treatment Clinical Trials Network (CTN) study “Optimizing Retention, Duration, and Discontinuation Strategies for Opioid Use Disorder Pharmacotherapy” (RDD; CTN-0100) will be among the largest clinical trials on treatment of OUD yet conducted, consisting of two phases, the Retention phase, and the Duration-Discontinuation phase. The Retention phase, open to patients initiating treatment, will test different doses and formulations of buprenorphine (standard dose sublingual, high dose sublingual, or extended-release injection), and a digital therapeutic app delivering contingency management and cognitive behavioral counseling on the primary outcome of retention in treatment. The Discontinuation phase, open to patients in stable remission from OUD and choosing to discontinue medication (including participants from the Retention phase or from the population of patients treated at the clinical site, referred by an outside prescriber or self-referred) will study different tapering strategies for buprenorphine (sublingual taper vs taper with injection buprenorphine), and a digital therapeutic app which provides resources to promote recovery, on the primary outcome of relapse-free discontinuation of medication. This paper describes how the RDD trial derives from two decades of research in the CTN. Initial trials (CTN-0001; CTN-0002; CTN-0003) focused on opioid detoxification, showing buprenorphine-naloxone was effective for detoxification, but that acute detoxification did not appear to be an effective treatment strategy. Trials on comparative effectiveness of medications for opioid use disorder (MOUD) (CTN-0027; CTN-0030; and CTN-0051) highlighted the problem of dropout from treatment and few trials defined retention on MOUD as the primary outcome. Long-term follow-up studies on those patient samples demonstrated the importance of long-term continuation of medication for many patients to sustain remission. Overall, these trials highlight the potential of a stable research infrastructure such as CTN to advance treatment effectiveness through a programmatic succession of large clinical trials.

## Background

Three medications have been approved for treatment of OUD by the Food and Drug Administration (FDA) [1]. Methadone has been used since 1964 in the United States in maintenance clinics providing observed, once-daily dosing [2]. Buprenorphine, originally developed for the treatment of pain, has been used since the 1990s internationally to treat opioid use disorder and was approved for OUD in the United States in 2002 after the Drug Addiction Treatment Act of 2000 (DATA 2000) was passed allowing outpatient providers to use buprenorphine for maintenance of opioid use disorder [3]. Buprenorphine is usually administered via daily sublingual dosing, but recently long-acting injectable formulations have been developed [4]. One of these formulations, Sublocade, has been approved for the treatment of opioid use disorder in the United States and is available for use clinically [5]. The other, Brixadi, has been approved for use in Europe and has received provisional approval in the United States [6]. Both buprenorphine and methadone are agonists at the opioid receptor. The third medication, naltrexone, developed in the 1970s and 1980s, is an opioid antagonist, blocking the effects of opioids at the mu opioid receptor. Although effective at decreasing drug use and overdose if taken daily, oral naltrexone has not been found to be effective in general clinical practice because of problems with adherence. It also is effective in improving alcohol use disorder outcomes and has been used extensively for that indication [7]. Long-acting injectable naltrexone, a once-monthly injection, was approved by the FDA in 2006 for the treatment of alcohol use disorder and in 2010 for opioid use disorder.

Despite these effective options for medication treatment of OUD, significant clinical challenges still exist in addressing the serious public health impact of opioids. Indeed, in the United States, more than 46,000 people died of an opioid overdose in 2018 [8]. Challenges include expanding access to medication for OUD and retaining individuals who begin medication for OUD in treatment.

The National Drug Abuse Clinical Trials Network (NIDA CTN) was established in 1999, bringing together a network of academic research centers in partnership with local community-based addiction treatment programs and with NIDA. In the words of Alan Leshner, Ph.D., Director of NIDA at the time, the goal was to make “science the foundation for improved drug abuse treatment throughout the Nation” by accelerating both the pace of clinical research and dissemination into community-based treatment [9].

The CTN is “a multi-site research project of behavioral, pharmacological, and integrated treatment interventions to determine effectiveness across a broad range of community-based treatment settings and diversified patient populations” [10].

Since its inception, the CTN has funded many important trials on OUD and other substance use disorder treatment. Recently, as part of the National Institutes of Health’s Helping to End Addiction Long-term (HEAL) initiative, the CTN was tasked to study strategies to improve retention of individuals on medication for OUD as well as to consider conditions under which it would be possible for patients to safely stop the use of medication. A collaborative team was assembled to plan the RDD study (CTN-0100), which builds on studies completed until this point by the CTN.

This paper reviews the relevant CTN studies and describes how these studies provide the background for the CTN-0100 study. This literature review is focused on CTN studies and is not meant to provide the full rationale for the design decisions made in the RDD study. A full description of the rationale and details of the RDD protocol is beyond the scope of this paper, and we will only provide a broad overview of the study’s design.

### The CTN and opioid use disorder treatment over time

Long-term treatment of OUD patients with medications has only relatively recently been accepted as best practice. Historically, OUD treatment was mostly geared toward rapid detoxification followed by psychosocial support, usually with 12-step mutual support programs, without use of medication to maintain abstinence. This approach contrasted with the evidence that many individuals with OUD benefited from long-term maintenance with methadone since its initial use in the 1960s [2] and the evidence that OUD, like other substance use disorders, is a chronic disease requiring long-term treatment [11]. Over time, the treatment of OUD in the United States has transitioned from one focused on detoxification and psychosocial support of abstinence to longer-term medication use to support recovery. These changes have been reflected in, and at times driven by, trials sponsored by the NIDA CTN. Table 1 summarizes the CTN studies on OUD treatment.

**Table 1:** Summary of CTN trials relevant to the RDD study

Author year	Design	Summary of findings
Studies on detoxification		
Ling et al. 2005 (CTN-0001)	12 sites, 113 adult OUD in-patients OUD individuals seeking short-term inpatient treatment were randomly assigned, in a 2:1 ratio favoring buprenorphine-naloxone, to a 13-day detoxification using open label buprenorphine-naloxone or clonidine	77% of in-patients assigned to the buprenorphine-naloxone condition achieved success compared to 22% of patients assigned to clonidine (p = 0.05)
Ling et al. 2005 (CTN-0002)	12 sites, 231 adult OUD outpatients OUD individuals seeking outpatient treatment were randomly assigned, in a 2:1 ratio favoring buprenorphine-naloxone, to a 13-day detoxification using open label buprenorphine-naloxone or clonidine	46 of the 157 (29%) outpatients assigned to the buprenorphine-naloxone condition achieved the treatment success criterion, compared to four of the 74 (5%) assigned to clonidine (p = 0.05)
Ling et al. 2009 (CTN-0003)	11 sites, 516 adult OUD outpatients Compared open label buprenorphine short (7 day) or long (28 day) taper schedules after a 1-month stabilization phase	At the end of the taper 44% of the 7-day taper group (n = 255) provided opioid-free urine specimens compared to 30% of the 28-day taper group (n = 261; p = 0.0007). There were no differences at the 1-month and 3-month follow-ups
Woody et al. 2008 (CTN- 0010)	6 sites, 152 OUD outpatients age 15–21 Patients were randomized to either 12-weeks of buprenorphine-naloxone (up to 24 mg per day for 9 weeks and tapered over weeks 10–12) or to a 14-day buprenorphine-naloxone detoxification	Individuals assigned to 12 weeks of buprenorphine (n = 78) showed significantly lower rates of positive urine toxicology for opioids at weeks 4 (61% vs 26% p < .001) and 8 (54% vs 23% (p = 0.01)) compared to individuals assigned to detoxification (n = 74) (. At week 12 the groups did not differ significantly (p = 0.18)
Studies on long-term maintenance outcomes		
Saxon et al. 2012 (CTN-0027)	8 sites, 1,269 adult OUD outpatients Patients presenting for outpatient treatment were randomized to 24 weeks of open label flexible dosed buprenorphine-naloxone or methadone	There were no significant differences in medications for liver impact Individuals assigned to methadone (n = 529) were significantly more likely (p < 0.0001) to have remained in treatment through week 24 (74% vs 46%) compared to those assigned to buprenorphine-naloxone (n = 740)
Hser et al. 2016 (CTN-0050)	7 sites, 1080 participants of the CTN-0027 trial Patients were followed up 2–8 years after randomization the initial CTN-0027 study Patients self-reported opioid use and treatment status and provided a urine toxicology test and an oral rapid HIV test	Overall mortality was similar between buprenorphine (5.8%) and methadone (3.6%) participants (P = 0.10). Opioid use at follow up was higher among participants randomized to buprenorphine (42.8%) compared to methadone (31.7%) (p < 0.01). Both buprenorphine-naloxone and methadone were associated with lower opioid use compared to no treatment. More individuals who achieved long-term abstinence from both heroin and other opioids were in treatment compared to the non-treatment group (63.5% vs 50.9%)
Weiss et al. 2011 (CTN-0030)	10 sites, 653 prescription OUD patients Randomized two-phase clinical trial using an adaptive treatment research design to determine efficacy buprenorphine-naloxone with different counseling intensities for patients on prescription opioids Phase 1: Brief treatment (phase 1) 2-week buprenorphine-naloxone stabilization, 2-week taper, 8-week post medication follow up randomized to either standard medical management (SMM) or standard medical management + opioid dependence counseling (SMM + ODC) Phase 2: Those who did not meet study-defined success outcome during phase 1 were restarted on 12 weeks of buprenorphine-naloxone treatment (without taper) and again provided either SMM or SMM + ODC	Phase 1: only 6.6% of patients met study-defined outcomes for successful treatment. There was no significant difference (p = 0.39) in outcomes between SMM and SMM + ODC Phase 2: 49.2% met study-defined criteria for success; there was no significant difference (p = 0.21) in outcomes between SMM and SMM + ODC A history of ever using heroin was associated with lower phase 2 success rates
Weiss et al. 2015 (CTN-0030a-3)	375 patients out of the 653 patients from the CTN-0030 study enrolled in a 42-month follow up study Telephone interviews were administered at 18, 30, and 42 months after trial enrollment	At month 42: 31.7% were abstinent from opioids and not on agonist therapy. 29.4% were on agonist therapy, but not dependent on opioids. 7.5% were using illicit opioids while on agonist therapy. 31.4% were using opioids without agonist therapy Lifetime heroin use was associated with opioid dependence at 42 months (p < 0.05). Agonist therapy was associated with greater likelihood of illicit opioid abstinence
Campbell et al. 2014 (CTN-0044)	10 sites, 507 patients with various substance use disorders, including individuals with OUD (n = 108) but no patients on medication for OUD Adults entering outpatient treatment were randomized to 12 weeks of treatment as usual or 12 weeks of treatment as usual and Therapeutic Education System (the therapeutic education system) The therapeutic education system consisted of 62 computer interactive modules with financial incentives	The therapeutic education system reduced dropout rate (p = 0.01) and increased abstinence rates (p = 0.01) by nearly double in individuals with non-opioid substance use disorders Individuals with OUD did not show improvement with the addition of the therapeutic education system
Lee et al. 2018 (CTN-0051)	8 sites, 570 adult OUD patients, recruited on inpatient detoxification and residential units Patients were randomized to either extended-release naltrexone (injectable naltrexone) OR sublingual buprenorphine-naloxone for outpatient maintenance treatment	Significantly fewer (p < 0.0001) individuals initiated XR—NTX (n = 204, 72%) vs buprenorphine-naloxone (n = 270, 94%) Among patients successfully inducted, 24-week relapse rates were similar (p = 0.44)

Reflecting standard practice in the late 1990s and early 2000s that treatment began with detoxification, the first three trials completed by the CTN tested opioid detoxification approaches. The first and second trials using the CTN framework considered the newly approved medication buprenorphine-naloxone for the facilitation of detoxification [12]. These trials compared a thirteen-day sublingual buprenorphine-naloxone taper with the centrally acting alpha-2 agonist clonidine for individuals with OUD in inpatient and outpatient levels of care. The primary outcome was defined as taper completion with urine-confirmed opioid abstinence at day 13. This outcome was chosen “because it reflects what clinicians ultimately consider important in treating (patients who use heroin), i.e., the success criterion reflects both retention and opioid abstinence” [12]. This primary end point of completing detoxification over a very short period reflected the goals of clinicians at the time. Both the inpatient and outpatient trials found clear superiority for a sublingual buprenorphine-naloxone taper compared to clonidine. Based on this finding, the third CTN trial compared two approaches to outpatient sublingual buprenorphine-naloxone taper to facilitate detoxification [13]. All participants were inducted onto buprenorphine-naloxone and maintained for 1 month. Participants were then randomized to two lengths of buprenorphine-naloxone taper, either a 7- or 30-day taper. The primary endpoint of this trial was again successful completion of the taper period with an opioid-free urine test at taper end and at 1- and 3-month outcome time points. A significantly higher percentage of individuals in the 7-day taper group presented with opioid-negative urine tests than those in the 30-day taper group. This finding may have been due to the differential time lapse before primary outcome (individuals had been stabilized for a month before beginning taper and 7 days may have been too short a period to lead to relapse). More significant was the extremely low percentage of the study population retained and with an opioid-negative urine test at one- (around 17%) and 3-month (around 12–14%) follow-up. This finding signaled the need for reconsideration of the goal of a relatively rapid taper off buprenorphine.

Due to increasing rates of OUD in young adults, the CTN conducted a trial testing buprenorphine maintenance treatment versus detoxification in adolescents and young adults (ages 14–21) with OUD (CTN-0010) [14]. Participants were randomized to either a sublingual buprenorphine-naloxone taper (induction and 2-week taper) or a 12-week course of sublingual buprenorphine-naloxone treatment (9 weeks of maintenance followed by a 3-week taper). These results again highlighted the benefits of a longer course of medication for OUD treatment. Individuals in the extended treatment group showed significantly lower rates of opioid-positive urine tests at weeks four (61% vs 26%) and eight (54% versus 23%). At week 12, the groups did not differ significantly, driven by a higher opioid-positive rate in the extended treatment group at the end of taper. The findings highlighted the clear benefit of longer medication for OUD treatment for this population and implied that even 9 weeks of maintenance treatment are insufficient. Unfortunately, few trials consider longer-term outcomes in this patient population.

With regard to the adult OUD population, the CTN also funded a definitive trial supporting the need for an extended course of treatment (CTN-0030) [15]. The Prescription Opioid Addiction Treatment Study (POATS) enrolled only individuals who were primarily or exclusively prescription opioid users, reflecting the surge in prescription opioid use in the mid to late 2000s. It was also theorized that psychosocial treatment might have a greater impact in this population. All participants initially received 2 weeks of sublingual buprenorphine-naloxone treatment followed by a 2-week sublingual buprenorphine-naloxone taper period. Individuals were randomized to receive either medical management alone or medical management plus individual opioid drug counseling during this initial 4-week period. Individuals in both arms of the trial showed poor chances of successful recovery, with 93% of participants returning to opioid use before 8 weeks following the taper period (i.e., week 12 overall). In a second phase of the trial, individuals who had relapsed to opioid use were restarted and stabilized on sublingual buprenorphine-naloxone maintenance and were re-randomized to the same psychosocial treatment conditions as during the taper phase. This phase showed that about 50% of individuals had successful opioid use outcomes while stabilized on buprenorphine-naloxone for 12 weeks, i.e., they abstained from opioids for at least 3 of the 4 final weeks of buprenorphine-naloxone treatment (weeks 9–12), including week 12. There were no significant difference between those who did and did not receive counseling in addition to medical management.

In light of this and other strong evidence for the benefits for longer-term treatment with medication, the research focus moved to identifying best approaches for maintaining individuals on MOUD. The CTN funded several important trials in this area, including two large pragmatic trials comparing the FDA-approved medications for long-term treatment. The CTN-0027 Starting Treating with Agonist Replacement Therapy (START) trial compared liver toxicity in oral methadone and sublingual buprenorphine-naloxone-treated study participants as a primary outcome but also included secondary outcomes of retention and opioid use [16]. This trial, conducted at outpatient opioid treatment programs licensed to dispense methadone, randomized individuals presenting for treatment to either methadone or sublingual buprenorphine-naloxone in a flexible dosing schedule. The trial demonstrated that the two medications had comparable medical safety profiles, and both had relatively little impact on liver function. Treatment retention was significantly higher in the group receiving methadone (74% versus 46%) at week 24. The trial also demonstrated a clear association between higher doses of both methadone and buprenorphine and retention. The trial used a symptom-driven dosing schedule with dose increases in response to cravings, withdrawal or opioid use. The mean maximum daily dose for buprenorphine was 22.1 mg and 91.2 mg for methadone [17]. Few trials have prospectively randomized participants to high- versus low-dose buprenorphine.

A second trial compared long-acting injectable naltrexone with sublingual buprenorphine with regard to relapse-free treatment (X-BOT; CTN-0051) [18]. This trial randomized individuals on inpatient detoxification and residential units to be inducted on either extended-release naltrexone or sublingual buprenorphine-naloxone. The primary outcome was defined as the length of successful treatment without relapse. Because of the differences in beginning patients on buprenorphine-naloxone versus injectable naltrexone, the trial found that it was significantly less likely patients would be inducted onto injectable naltrexone, leading to superior outcome overall of buprenorphine. However, in an analysis of only individuals who were inducted onto their assigned medication, the two medications had comparable outcomes in terms of time-to-relapse.

The findings that psychosocial intervention without medication for OUD are not effective in the OUD population were replicated in the CTN-0044 trial testing the efficacy of an automated psychosocial intervention, the Therapeutic Education System, in individuals seeking treatment for a range of substance use disorders [19]. The intervention included automated therapy lessons combined with contingency management rewards provided for completion of lessons and negative urine toxicology. Individuals across substances other than opioids who received usual treatment plus the therapeutic education system were significantly more likely to be abstinent over the final weeks of the trial. Trial participants with OUD (none of whom was on medication for OUD) did not benefit from the therapeutic education system. In contrast, trials conducted outside of the CTN, testing the therapeutic education system along with medication for OUD, have shown benefit of the intervention [20–22].

Long-term follow up trials after CTN-0027 [23] and CTN-0030 [24] showed that individuals who remained on medication for OUD had significantly less opioid use than those who discontinued medication treatment. Both trials also found that over time most participants improved in terms of severity of drug use regardless of treatment status. A significant proportion of individuals, ranging from about one-third to one-half of the study population, were able to achieve periods of abstinence without medication at later follow-up time points [24].

The above trials highlighted the difficulty in maintaining OUD patients in treatment. In the START trial (CTN-0027), comparing buprenorphine-naloxone and methadone, only 46% of individuals maintained on buprenorphine-naloxone were maintained in treatment through the end of the 24-week trial. Although retention rates for individuals on methadone were higher (74%), these were still not optimal, with about 1 in 4 participants dropping out of treatment. Methadone is also difficult for many patients to access due to regulatory issues and the need to frequently present to a program dispensing methadone to receive care [25]. The Extended-Release Naltrexone vs. Buprenorphine for Opioid Treatment (X:BOT) trial (CTN-0051), comparing injectable naltrexone and sublingual buprenorphine, was not designed to capture data on retention (irrespective of clinical outcome), as time to relapse was the study's primary outcome. However, a 36-week follow up assessment for all randomized participants found that only 52% of participants who were assessed reported being maintained on medication [26]. This likely underestimated the number of individuals not continuing medication for OUD, as intensive efforts were made to assess all participants and individuals not assessed were likely no longer connected with treatment. Other clinical trials have similarly shown that 50% or more of participants drop out of treatment by 3 to 6 months after treatment initiation [27, 28]. New approaches to assist in maintaining individuals in treatment for a sufficient length of time to maintain recovery in the long term are therefore needed.

### CTN-0100: optimizing retention, duration, and discontinuation (rdd) trial

Evidence exists for numerous approaches to increasing retention to MOUD, [29] but few trials have prospectively tested alternate approaches, using retention as a primary outcome. To address the optimal approach to improve retention on medication for OUD, the CTN has planned the CTN-0100 study, a large, prospective, pragmatic, multisite trial. The first phase of the trial will focus on issues relating to retention in maintenance treatment for individuals prescribed buprenorphine or injectable naltrexone, with the primary outcome of achieving a minimum of 6 months maintenance on medication for OUD. Two clinical approaches will be tested in individuals choosing buprenorphine maintenance treatment, using a 3 × 2 factorial design randomization approach. The trial will address medication dose and formulation by randomization to one of three buprenorphine conditions: standard dose (target of 16 mg/day), high dose (target of 32 mg/day), or long-acting injectable buprenorphine. To address the benefit of an automated psychosocial intervention, all patients will also be independently randomized to usual psychosocial treatment or to usual treatment plus an automated psychosocial intervention (Pear-002a, an investigational version of reSET-O).

**Fig. 1:**
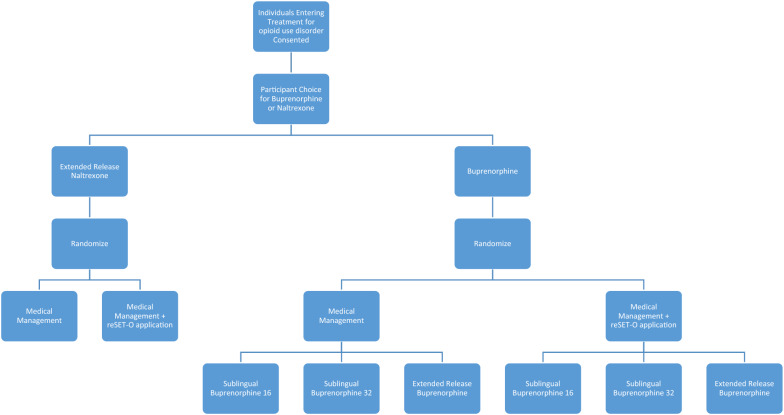
Retention phase

Participants choosing to be maintained on injectable naltrexone will not be randomized to any pharmacotherapy condition, but will be randomly assigned to one of the two behavioral interventions, usual psychosocial and medication management treatment with the automated psychosocial intervention (Pear-002a) or usual psychosocial and medication management alone. Figure 1 outlines the retention phase of the trial.

### Optimal length of treatment

In addition to issues of improving maintenance rates to medication for OUD, there is little evidence on the optimal timing of and best clinical practice for the discontinuation of medication for OUD. Importantly, there are no prospective data that can help clinicians advise patients regarding the likelihood that they will be able to safely discontinue medication without returning to opioid use. Although prospective randomized trials are not available, many non-randomized trials have used data from national registries and insurance claims to gather outcome data on large cohorts of individuals. Data from these registries imply that longer treatment courses (more than a year or two) are associated with better outcomes. For example, Eastwood et al. [30] reviewed data from all patients seeking treatment in England through a national registry and were able to follow outcomes over years. They found that individuals with two years of consecutive treatment had higher likelihood of achieving positive clinical outcomes for 6 months following the end of treatment. Similarly, a recently created Medicaid claims database of OUD patients who had discontinued buprenorphine after least 6 months of treatment [31] showed that individuals maintained for the longest time period (15–18 months) had significantly lower rates of emergency department visits, hospitalizations, and receipt of opioid prescriptions based on claims data. The authors noted that although the rates of these types of events were lower in the cohort who remained on medications for the longest time period, adverse events were still common in this cohort. In addition, the likelihood of a medical overdose was not significantly lower in individuals who had remained on medication for a longer period, strengthening the argument for longer or indefinite maintenance courses.

To address the question of whether individuals can be safely tapered off medication for OUD after an extended period of stabilization, the second study phase of CTN-0100 will follow stable individuals with OUD who attempt to stop their medication for OUD. Because of the risks of discontinuing medications, participants will not be actively recruited for the Discontinuation Phase and will not be randomized to a predetermined length of treatment. Instead, study participation will only be offered to individuals who (1) have been maintained for an extended period (buprenorphine [extended-release or sublingual] for at least one-year, injectable naltrexone for at least 6 months), (2) are currently stable in terms of abstaining from opioid and other drug use, and (3) wish to discontinue medication for OUD. The primary endpoint of the study is successfully stopping medications and not relapsing to opioid use within the 6 months following discontinuation. Individuals will be encouraged to remain on their medications if needed and continuation on MOUD without relapse will also be considered a positive outcome. All patients will be followed for the duration of the taper (a maximum of 24 weeks for participants entering on extended-release naltrexone and 48 weeks for those entering on buprenorphine) and at least 24 weeks following the end of the taper period. Predictive analysis will be used to determine which patients were most likely to be able to successfully complete taper of medication and be maintained without relapse to opioid use. In this way, data may be safely gathered on the likelihood of successful and safe taper without relapse.

**Fig. 2:**
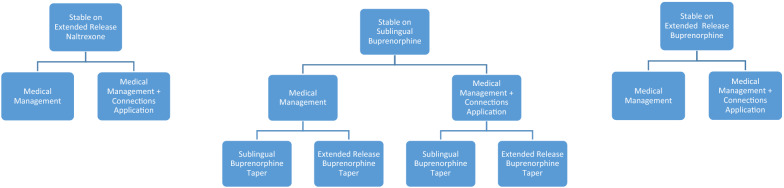
Discontinuation phase

In addition, this phase will also test approaches to facilitate the safe taper of medication. Automated psychosocial intervention in supporting patients in stopping their medication will be tested. All participants will be randomized to receive either usual treatment or usual treatment along with access to a recovery support app (the CHESS Connections App) [32, 33]. This application was chosen for the purpose of relapse prevention in this group, (as opposed to the Pear-002a application) because it includes tools specifically designed to support maintenance of sobriety during high-risk situations. Patients stopping sublingual buprenorphine will also be randomized to taper their sublingual dose of medication or to be transitioned to long-acting injectable buprenorphine to facilitate their medication taper. Figure 2 shows a diagram of the discontinuation phase.

## Conclusion

Treatment for OUD has shifted over time from acute withdrawal management to long term MOUD. Although medications have been found to be effective at assisting patients decrease opioid use and overdose, long-term retention of patients on these medications has been a significant challenge. In addition, the question of whether, how, and for whom these medications can be safely stopped after an extended period of stability has also not been addressed with prospective trials. The CTN-0100 trial will address some of these clinical issues using a two-phase, large, multisite, prospective, pragmatic trial.

The benefits of medications for long-term maintenance of OUD has also highlighted the importance of providing these treatments for all individuals with OUD. A significant challenge remains as most patients with OUD never begin treatment with medication for OUD [34]. To address this gap, the CTN has sponsored several trials to implement buprenorphine in medical settings including general emergency departments and primary care centers to increase the number of patients who may be offered and initiated onto medication for OUD.

The CTN is an example of a stable research infrastructure for carrying out large, definitive multi-site trials in the area of OUD along with other substance use disorders. In the coming years CTN trials will help address critical treatment issues and hopefully turn the page on the opioid and overdose crises that continue to have tragic consequences in the United States and around the world.

## Data Availability

Not applicable.

## Abbreviations

OUD: Opioid use disorder
CTN: Clinical Trials Network
RDD: Optimizing Retention, Duration, and Discontinuation Strategies for Opioid Use Disorder Pharmacotherapy
MOUD: Medications for opioid use disorder
FDA: Food and drug administration
NIDA CTN: National Institute on Drug Abuse Clinical Trials Network
NIDA: National Institute on Drug Abuse
NIH: National Institutes of Health
HEAL: Helping to End Addiction Long-term
POATS: Prescription Opioid Addiction Treatment Study
START: Starting Treatment with Agonist Replacement Therapy
SMM: Standard Medical Management
ODC: Opioid Dependence Counseling
XBOT: Extended-Release Naltrexone vs. Buprenorphine for Opioid Treatment

## References

[1] Volkow ND, Jones EB, Einstein EB, Wargo EM (2019). Prevention and treatment of opioid misuse and addiction: a review. JAMA Psychiatry..

[2] Kreek MJ (2000). Methadone-related opioid agonist pharmacotherapy for heroin addiction. History, recent molecular and neurochemical research and future in mainstream medicine. Ann N Y Acad Sci..

[3] Campbell ND, Lovell AM (2012). The history of the development of buprenorphine as an addiction therapeutic. Ann N Y Acad Sci.

[4] Haight BR, Learned SM, Laffont CM, Fudala PJ, Zhao Y, Garofalo AS (2019). Efficacy and safety of a monthly buprenorphine depot injection for opioid use disorder: a multicentre, randomised, double-blind, placebo-controlled, phase 3 trial. Lancet Lond Engl.

[5] Knopf A (2019). Sublocade trial data published: better than placebo. Alcohol Drug Abuse Wkly.

[6] Tiberg F. US FDA issues a tentative approval of Brixadi^TM^ (buprenorphine) extended-release injection for treatment of opioid use disorder. Camurus; 2018. https://mb.cision.com/Main/13456/2715933/975197.pdf

[7] O’Brien CP, Volpicelli LA, Volpicelli JR (1996). Naltrexone in the treatment of alcoholism: a clinical review. Alcohol Fayettev N.

[8] Overdose Death Rates. NIDA. 2020. https://www.drugabuse.gov/related-topics/trends-statistics/overdose-death-rates

[9] NIDA. NIDA’s Clinical Trials Network Marks Progress Toward Improved Drug Abuse Treatment. 2002; https://archives.drugabuse.gov/news-events/nida-notes/2002/02/nidas-clinical-trials-network-marks-progress-toward-improved-drug-abuse-treatment

[10] National Institute on Drug Abuse. Center for Clinical Trials Network (CCTN). DrugAbuse.gov. 2011. https://www.drugabuse.gov/about-nida/organization/center-clinical-trials-network-cctn

[11] Hser Y-I, Anglin MD, Grella C, Longshore D, Prendergast ML (1997). Drug treatment careers A conceptual framework and existing research findings. J Subst Abuse Treat.

[12] Ling W, Amass L, Shoptaw S, Annon JJ, Hillhouse M, Babcock D (2005). A multi-center randomized trial of buprenorphine-naloxone versus clonidine for opioid detoxification: findings from the National Institute on Drug Abuse Clinical Trials Network. Addict Abingdon Engl.

[13] Ling W, Hillhouse M, Domier C, Doraimani G, Hunter J, Thomas C (2009). Buprenorphine tapering schedule and illicit opioid use. Addict Abingdon Engl.

[14] Woody GE, Poole SA, Subramaniam G, Dugosh K, Bogenschutz M, Abbott P (2008). Extended vs short-term buprenorphine-naloxone for treatment of opioid-addicted youth: a randomized trial. JAMA.

[15] Weiss RD, Potter JS, Fiellin DA, Byrne M, Connery HS, Dickinson W (2011). Adjunctive counseling during brief and extended buprenorphine-naloxone treatment for prescription opioid dependence: a 2-phase randomized controlled trial. Arch Gen Psychiatry.

[16] Saxon AJ, Ling W, Hillhouse M, Thomas C, Hasson A, Ang A (2013). Buprenorphine/Naloxone and methadone effects on laboratory indices of liver health: a randomized trial. Drug Alcohol Depend.

[17] Hser Y-I, Saxon AJ, Huang D, Hasson A, Thomas C, Hillhouse M (2014). Treatment retention among patients randomized to buprenorphine/naloxone compared to methadone in a multi-site trial. Addict Abingdon Engl.

[18] Lee JD, Nunes EV, Novo P, Bachrach K, Bailey GL, Bhatt S (2018). Comparative effectiveness of extended-release naltrexone versus buprenorphine-naloxone for opioid relapse prevention (X:BOT): a multicentre, open-label, randomised controlled trial. Lancet Lond Engl..

[19] Campbell ANC, Nunes EV, Matthews AG, Stitzer M, Miele GM, Polsky D (2014). Internet-delivered treatment for substance abuse: a multisite randomized controlled trial. Am J Psychiatry.

[20] Bickel WK, Marsch LA, Buchhalter AR, Badger GJ (2008). Computerized behavior therapy for opioid-dependent outpatients: a randomized controlled trial. Exp Clin Psychopharmacol.

[21] Christensen DR, Landes RD, Jackson L, Marsch LA, Mancino MJ, Chopra MP (2014). Adding an Internet-delivered treatment to an efficacious treatment package for opioid dependence. J Consult Clin Psychol.

[22] Marsch LA, Guarino H, Acosta M, Aponte-Melendez Y, Cleland C, Grabinski M (2014). Web-based behavioral treatment for substance use disorders as a partial replacement of standard methadone maintenance treatment. J Subst Abuse Treat.

[23] Hser Y-I, Evans E, Huang D, Weiss R, Saxon A, Carroll KM (2016). Long-term outcomes after randomization to buprenorphine/naloxone versus methadone in a multi-site trial. Addict Abingdon Engl.

[24] Weiss RD, Potter JS, Griffin ML, Provost SE, Fitzmaurice GM, McDermott KA (2015). Long-term outcomes from the National Drug Abuse Treatment Clinical Trials Network Prescription Opioid Addiction Treatment Study. Drug Alcohol Depend.

[25] Yarborough BJH, Stumbo SP, McCarty D, Mertens J, Weisner C, Green CA (2016). Methadone, buprenorphine and preferences for opioid agonist treatment: a qualitative analysis. Drug Alcohol Depend.

[26] Greiner M. Medication Status, Opioid Abstinence, and Relapse Following the X:BOT Trial. Oral Presentation presented at: CPDD Annual Meeting; 2020; College on Problems of Drug Dependence.

[27] Liebschutz JM, Crooks D, Herman D, Anderson B, Tsui J, Meshesha LZ (2014). Buprenorphine treatment for hospitalized, opioid-dependent patients: a randomized clinical trial. JAMA Intern Med.

[28] Fiellin DA, Barry DT, Sullivan LE, Cutter CJ, Moore BA, O’Connor PG (2013). A randomized trial of cognitive behavioral therapy in primary care-based buprenorphine. Am J Med.

[29] Timko C, Schultz NR, Cucciare MA, Vittorio L, Garrison-Diehn C (2016). Retention in medication-assisted treatment for opiate dependence: a systematic review. J Addict Dis.

[30] Eastwood B, Strang J, Marsden J (2017). Effectiveness of treatment for opioid use disorder: a national, five-year, prospective, observational study in England. Drug Alcohol Depend.

[31] Williams AR, Samples H, Crystal S, Olfson M (2020). Acute care, prescription opioid use, and overdose following discontinuation of long-term buprenorphine treatment for opioid use disorder. Am J Psychiatry.

[32] Gustafson DH, Bosworth K, Hawkins RP, Boberg EW, Bricker E. CHESS: a computer-based system for providing information, referrals, decision support and social support to people facing medical and other health-related crises. Proc Symp Comput Appl Med Care. 1992. pp. 161–5. PMC2248029 1482860

[33] Gustafson DH, Hawkins RP, Boberg EW, McTavish F, Owens B, Wise M (2002). CHESS: 10 years of research and development in consumer health informatics for broad populations, including the underserved. Int J Med Inf.

[34] Williams AR, Nunes EV, Bisaga A, Levin FR, Olfson M (2019). Development of a Cascade of Care for responding to the opioid epidemic. Am J Drug Alcohol Abuse.

